# Lack of N-glycosylation increases amyloidogenic processing of the amyloid precursor protein

**DOI:** 10.1093/glycob/cwac009

**Published:** 2022-03-10

**Authors:** Tong Lin, Lea S van Husen, Yang Yu, Lars O Tjernberg, Sophia Schedin-Weiss

**Affiliations:** Division of Neurogeriatrics, Department of Neurobiology, Care Sciences and Society, Karolinska Institutet, BioClinicum, J9:20, Visionsgatan 4, Stockholm 171 64, Sweden; Division of Neurogeriatrics, Department of Neurobiology, Care Sciences and Society, Karolinska Institutet, BioClinicum, J9:20, Visionsgatan 4, Stockholm 171 64, Sweden; Division of Neurogeriatrics, Department of Neurobiology, Care Sciences and Society, Karolinska Institutet, BioClinicum, J9:20, Visionsgatan 4, Stockholm 171 64, Sweden; Division of Neurogeriatrics, Department of Neurobiology, Care Sciences and Society, Karolinska Institutet, BioClinicum, J9:20, Visionsgatan 4, Stockholm 171 64, Sweden; Division of Neurogeriatrics, Department of Neurobiology, Care Sciences and Society, Karolinska Institutet, BioClinicum, J9:20, Visionsgatan 4, Stockholm 171 64, Sweden

**Keywords:** Aβ42, Alzheimer disease, amyloid precursor protein, N-glycosylation, processing and trafficking

## Abstract

The amyloid precursor protein (APP) is a ubiquitously expressed type 1 transmembrane protein mostly known for serving as a precursor to the amyloid-β peptide (Aβ), a culprit in Alzheimer disease (AD). However, APP also has important physiological functions by being implicated in, for instance, adhesion, signaling, neuronal development, and synaptic function. Human APP contains 2 N-glycosylation sites, at asparagine (N) 467 (N467) and N496. Here, we studied the role of N-glycosylation on APP trafficking and processing by constructing APP-SNAP plasmid vectors for wildtype APP and N-glycosylation site mutants in which N467 or N496 was replaced by glutamine (Q) and expressed these in HEK293T cells. Lack of either of the 2 *N*-glycans resulted in a reduction in the size of intracellular APP-SNAP-positive vesicles and a reduction of APP-SNAP in the plasma membrane and lysosomes. Importantly, loss of either of the 2 *N*-glycans resulted in elevated levels of intracellular as well as secreted Aβ42. These data suggest that *N*-glycans have a major impact on trafficking and processing of APP and could play an important role in the development of AD.

## Introduction

The amyloid precursor protein (APP) is expressed in most cells, both in brain and in periphery. It is a type I transmembrane glycoprotein, mostly known for its role as a precursor for the neurotoxic amyloid β-peptide (Aβ), a causative agent in Alzheimer disease (AD) (Selkoe and Hardy 2016). However, it is also implicated in several physiological functions, including cell adhesion and signaling, and plays crucial roles for neuronal functions by regulating for instance neurogenesis and synaptic plasticity (Coulson et al. 2000; Muller et al. 2017). APP is encoded by a single gene and is subjected to differential splicing. In humans, the major variants contain 695, 751, or 770 amino acids. The variant with 695 amino acids (APP695) is the predominant form in neurons.

APP, containing a short C-terminal intracellular domain and a large extracellular/lumenal domain, is processed by different combinations of transmembrane aspartyl proteases, which leads to the generation of several fragments. In the nonamyloidogenic pathway, APP is first processed by ⍺-secretase to generate soluble APPα (sAPPα) and the membrane-bound C-terminal fragment α (CTFα), followed by γ-secretase cleavage of CTFα to generate a short fragment called P3 and APP intracellular domain (AICD). In the amyloidogenic pathway, the first cleavage is mediated by β-secretase to generate soluble APPβ and CTFβ, followed by γ-secretase cleavage of CTFβ, which generates AICD and the 40–43 residues long Aβ (Sisodia 1992; Yuksel and Tacal 2019). The most abundant Aβ peptide contains 40 amino acids (Aβ40), while the most aggregation-prone Aβ isoforms contain 42 or 43 amino acids (Aβ42 or Aβ43) (Radde et al. 2006; Welander et al. 2009). These species can polymerize into toxic aggregates and are significantly increased in AD brains (Welander et al. 2009; Sandebring et al. 2013). Current concepts suggest that APP and its processed products are dynamically transported within distinct vesicles along secretory, endocytic, and recycling routes (Thinakaran and Koo 2008; Choy et al. 2012; Haass et al. 2012). Since the APP transport route determines its processing fate, alterations in APP transport can directly influence whether APP releases nontoxic peptides or the neurotoxic Aβ. Dysregulation in cellular trafficking of APP could thus result in AD (Lin et al. 2021). It has been reported that APP cannot be processed before it has matured by cotranslational modifications in the endoplasmic reticulum (ER) and posttranslational modifications in the Golgi apparatus (Tomita et al. 1998; Greenfield et al. 1999). Thus, alterations in the co- and posttranslational modifications could result in a shift in APP transportation and processing (Yazaki et al. 1996; Da Cruz E Silva et al. 2004; Agostinho et al. 2015).

Intriguingly, N-glycosylation profiles are altered in AD patients (Schedin-Weiss et al. 2014, 2020; Gaunitz et al. 2020), including the *N*-glycan pattern on APP (Akasaka-manya et al. 2010; Palmigiano et al. 2016; Frenkel-Pinter et al. 2017; Cho et al. 2019; Tao and Huang 2019; Boix et al. 2020; Zhang et al. 2020). Hence, elucidating how N-glycosylation affects APP processing and trafficking will shed light on the physiological and pathological roles of APP N-glycosylation. N-glycosylation defects caused by glycosylation inhibitors have been shown to result in improper maturation, processing, and trafficking of APP. For example, cellular trafficking of APP to the cell surface (McFarlane et al. 1999), as well as to the axonal synaptic membrane (McFarlane et al. 2000), was altered when cells were treated with the ⍺-mannosidase inhibitor 2-deoxymannojirimycin, which specifically blocks the formation of hybrid and complex glycans. It has also been suggested that improper maturation resulting from lack of glycosylation leads to APP accumulation within the perinuclear region (Tienari et al. 1996; McFarlane et al. 1999). It should be noted, though, that such treatments affect glycosylation of also other proteins and, thus, it is difficult to draw conclusions specifically about the effects of the glycans attached to APP.

hAPP695 contains 2 N-glycosylation sites, asparagine (N) 467 (N467) and N496. There are no additional N-glycosylation sites in the longer APP splice variants. The N-linked oligosaccharides of APP have been suggested to be of the bi- or triantennary complex types with a fucosylated trimannosyl core containing terminal sialic acid residues (Påhlsson et al. 1992; Dichgans et al. 1993; Saito et al. 1995). It has been reported that there is a delayed sorting and, thus, an intravesicular accumulation of N467A or N496A mutant APP (Yazaki et al. 1996). Furthermore, the N467A or N496A mutations caused reduced APP levels at the cell surface and reduced level of secreted soluble APP (Tsatsanis et al. 2019). Still, many details of the role of APP glycosylation remain to be clarified.

Here, we studied the role of N-glycosylation on APP processing and trafficking by using vectors for expression of wildtype (WT) or N-glycosylation site mutants of hAPP695-SNAP (hereafter referred to as APP-SNAP) into HEK293T cells. We showed that the replacement of N by Q at either of the 2 N-glycosylation sites of APP-SNAP resulted in an overall reduction in the size of APP-SNAP-positive vesicles and reduced levels of APP in the plasma membrane and lysosomes. Importantly, the N-glycosylation mutations lead to increased levels of both intracellular and secreted Aβ42, suggesting that the removal of either of the 2 *N*-glycans results in enhanced amyloidogenic processing.

## Results

### Determination of APP-SNAP expression level

**Fig. 1:**
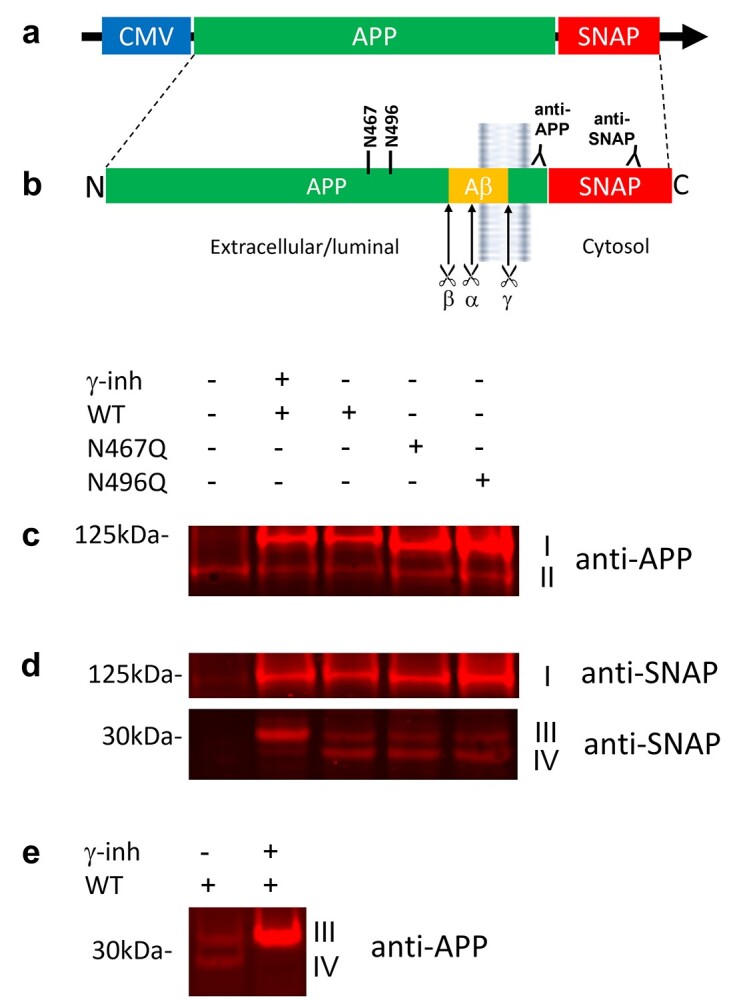
Expression of APP-SNAP variants in HEK293T cells. HEK293T cells were transfected with plasmids to express the APP-SNAP variants WT, N467Q, or N496Q and cultured for 48 h posttransfection. a) Schematic image of the DNA sequence for APP-SNAP, with a CMV promotor. b) Schematic image of the resulting APP-SNAP protein variants displaying the N-glycosylation sites (N467 and N496), the α-, β-, and γ-secretase cleavage sites and the antibody binding sites for the anti-APP (Y188) and anti-SNAP antibodies. To confirm the identity of the bands at ~30 kDa, cells expressing WT APP-SNAP were treated with the γ-secretase inhibitor L-685,458 (γ-inh). The cells were lysed and the lysate was subjected to SDS-PAGE followed by immunoblotting with an anti-APP antibody (Y188) (c) or an anti-SNAP-antibody (d). Band I at ~125 kDa represents full-length APP-SNAP and band II at ~100 kDa represents endogenous APP. The CTFs of APP-SNAP were located at ~30 kDa (d and e). Band III represents a combination of C83/C99 and band IV represents AICD.

DNA vectors for expression of APP-SNAP protein variants with or without mutations in the N-glycosylation sites (Fig. 1a and b) were generated as described in Materials and methods. The expression levels were variable for both the WT and N-glycosylation mutants and excessive overexpression was accompanied by retention of APP-SNAP in the ER, both for WT and N-glycosylation mutants (Fig. S1). To avoid excessive overexpression of the APP variants, the expression levels were therefore optimized (Fig. S2).

The identity of APP-SNAP, and the proteolytic fragments resulting from APP processing in HEK293T cells, was confirmed by western blotting probed with anti-APP (Y188) and anti-SNAP-tag antibodies (Fig. 1c–e). The band at ~125 kDa (Fig. 1c and d, band I) corresponds to full-length APP (~100–110 kDa) conjugated to a SNAP-tag (19.4 kDa). The absence of APP-SNAP in the nontransfected control (NT) was confirmed (Fig. 1d, left lane) and the expression of endogenous APP in HEK293T cells appeared as an additional band at ~100 kDa when probed with anti-APP antibody (Fig. 1c). The levels of endogenous APP were lower as compared with APP-SNAP (Fig. 1c and d). As expected, there was a slight decrease in the molecular weight of the full-length APP band for the N467Q and N496Q mutants (Fig. 1c and d), supporting that both these N-glycosylation sites are occupied by an *N*-glycan in the WT. Two additional bands at ~30 kDa (Fig. 1d and e, bands III and IV) were shown to represent the processed APP C-terminal fragments (CTFs); one corresponding to C99-SNAP/C83-SNAP (Fig. 1e, band III) and one corresponding to APP intracellular domain (AICD-SNAP; Fig. 1e, band IV). This was confirmed by treatment of the HEK293T cells expressing WT APP-SNAP with a γ-secretase inhibitor (L685,485) right after the transfection, which resulted in nondetectable levels of the AICD-SNAP band (Fig. 1e, band IV). Altogether, these experiments showed that the expression of full-length APP with a SNAP-tag and the processing of APP-SNAP was successful in HEK293T cells.

### APP-SNAP is mainly observed in intracellular vesicles

**Fig. 2:**
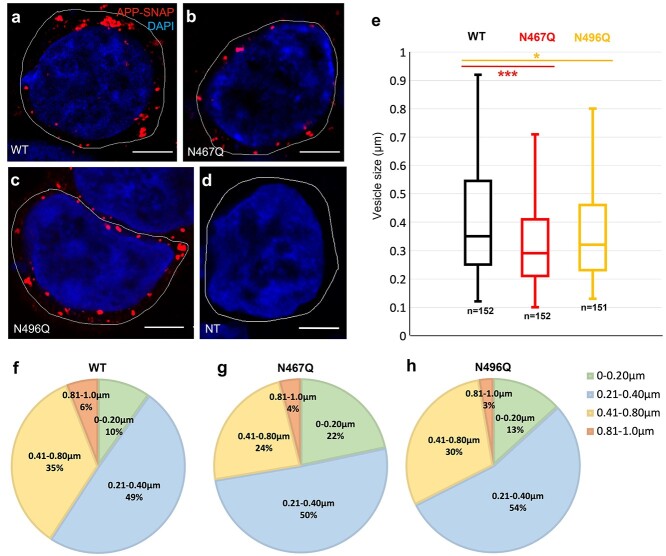
Size distribution and expression of APP-SNAP-positive vesicles in HEK293T cells. The size of APP-SNAP-containing vesicles was compared for the N-glycosylation mutants and the WT, 48 h after transfection of HEK293T cells. The cells were fixed, permeabilized, immunolabelled, and imaged by confocal microscopy. Cells with moderate expression were chosen for analysis to avoid formation of enlarged lysosomes caused by overexpression, as described in Materials and methods. The SNAP-tag of APP was labelled with TMR-star (red) and DAPI was used to stain nuclei (blue). Representative confocal images (a–d) are shown for WT (a), N467Q (b), N496Q (c), and NT (d). Scale bars: 5 μm. The size of each APP-SNAP-positive vesicle within the selected cells was analyzed using Fiji/image J and the size distribution is shown in (e). The results are based on at least 3 independent experiments (each from a different HEK293T cell passage) and *n* is the number of APP-SNAP-positive vesicles. Significance was calculated using *t*-test with equal variance; ^*^, 0.01 < *P* < 0.05; ^***^, 0.0005 < *P* < 0.005; n.s., nonsignificant. The distribution was also analyzed in subpopulations of vesicles with different sizes: 0–0.20, 0.21–0.40, 0.41–0.80, and 0.81–1.0 μm for WT (f), N467Q (g), and N496Q (h) variants.

**Fig. 3:**
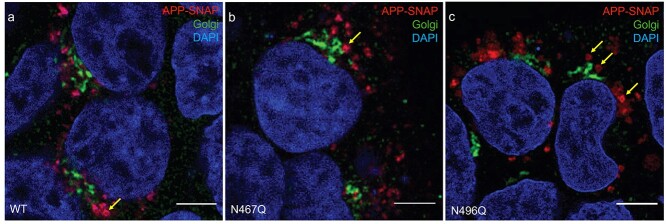
Localization of APP-SNAP variants in proximity to the Golgi apparatus. Confocal images of APP-SNAP variants in proximity to the Golgi apparatus, imaged 48 h after transfection of HEK293T cells. Representative images for WT (a), N467QA (b), and N496Q (c) are shown. Anti-GRASP65 antibody was used to stain the Golgi apparatus, while the SNAP-tag of APP was labelled with TMR-star (red) and DAPI was used to stain nuclei (blue). Yellow arrows point at vesicles that are sufficiently resolved to show the location of APP in the vesicular membrane. Scale bars: 5 μm.

The staining of APP-SNAP was, when excessive overexpression was avoided, to a large extent localized to intracellular compartments with morphologies that are consistent with vesicles along the endosomal/lysosomal pathway (Fig. 2a–c). Since the SNAP-tag is located in the C-terminus, the SNAP-tag labeling can represent full-length APP as well as APP-CTF. There was no SNAP-tag labeling in nontransfected (NT) controls (Fig. 2d), which confirmed that there was no unspecific binding of the fluorescence reagent (SNAP-Cell TMR-Star) in these cells. Most of the APP-SNAP-positive vesicles ranged in sizes with a diameter of up to 1 μm. There was a decrease in the size of the vesicles containing APP-SNAP for the N-glycosylation mutants (Fig. 2e), which was more significant in the cells expressing the N467Q mutant than those expressing the N496Q mutant. We further divided the vesicles into 4 groups based on their size: 0–0.20 μm, 0.21–0.40 μm, 0.41–0.80 μm, and 0.81–1.0 μm. There was no significant difference between WT and the mutants when subdivided into these smaller groups, possibly reflecting the lower number of vesicles per group (Fig. 2f–h). As expected, since APP is a type I transmembrane protein, it was located in the membrane of the vesicles (Fig. 3).

### Staining of APP-SNAP in proximity to the Golgi apparatus

Since the Golgi apparatus is involved in processing and elongation of the *N*-glycans of proteins, we stained the transfected HEK293T cells with a Golgi marker to study whether the N-glycosylation variants of APP accumulated in the Golgi apparatus. In the majority of the cells, there was only little colocalization between any of the APP variants and the Golgi marker GRASP65, a protein tightly connected to the Golgi membranes (Fig. 3), although APP vesicles were present close to the Golgi apparatus.

### APP-SNAP distribution in the plasma membrane

**Fig. 4:**
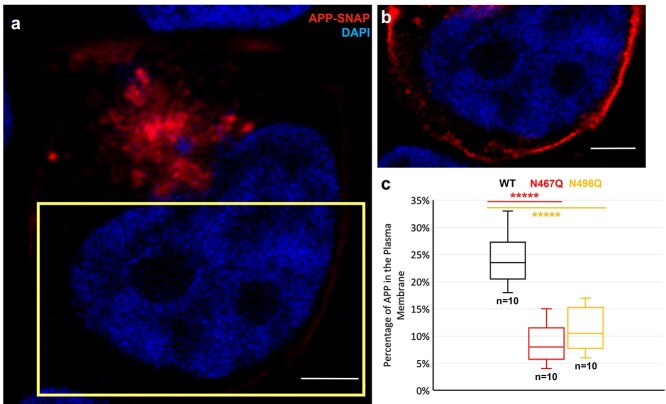
Proportion of APP-SNAP in the plasma membrane of HEK293T cells. To analyze the extent of presence of APP-SNAP in the plasma membrane, 102 of the HEK293T cells with a moderate level of APP-SNAP expression 48 h posttransfection were selected from confocal images from at least 3 independent experiments (each from a different HEK293T cell passage). The intensity of APP-SNAP in the plasma membrane was considerably weaker than that present in intracellular vesicles, requiring different settings upon image analysis in Fiji/image J (a and b). The area within the yellow rectangle in (a) is shown at higher fluorescence intensity in (b). Scale bars, 5 μm. The presence of APP-SNAP in the plasma membrane could only be detected for 35.3% (wildtype, WT), 11.8% (N467Q), and 9.8% (N496Q) of the cells in each group. Among the cells for which APP-SNAP could be detected in the plasma membrane, there was significantly lower extent of APP-SNAP in the plasma membrane in the cells expressing N-glycosylation mutants compared with the WT (Fig. 3c). The relative amount of APP-SNAP in the plasma membrane was calculated by subtracting (1) the total intensity of APP-SNAP by (2) the intensity of intracellular APP-SNAP in each cell. The results were then further calculated in percentage and plotted in a box and whisker graph (c). *N* is the number of HEK293T cells. Data significance was calculated using *t*-test with equal variance; ^*****^; *P* < 0.00005.

APP-SNAP was detected in the plasma membrane for some, but not all, of the transfected cells, albeit with a lower signal intensity than for the intracellular vesicles (Fig. 4a and b). The cells expressing the mutated N-glycosylation variants of hAPP695 displayed a lower proportion of cells for which we could detect APP-SNAP in the plasma membrane as compared with the WT. To be more specific, 102 of the HEK293T cells with a moderate level of APP-SNAP expression were selected for this analysis, among which 35.3% (WT), 11.8% (N467Q), and 9.8% (N496Q) contained APP-SNAP expression in the plasma membrane. Moreover, among the cells for which we detected APP-SNAP in the plasma membrane, the relative amount of mutant APP-SNAP in the plasma membrane compared with the total intensity of the APP-SNAP in each cell was significantly lower for the N-glycosylation mutants than for the WT (Fig. 4c). Thus, lack of N-glycosylation results in altered transport and/or processing of APP.

### About 30% of APP-SNAP showed an apparent colocalization with early endosomes

**Fig. 5:**
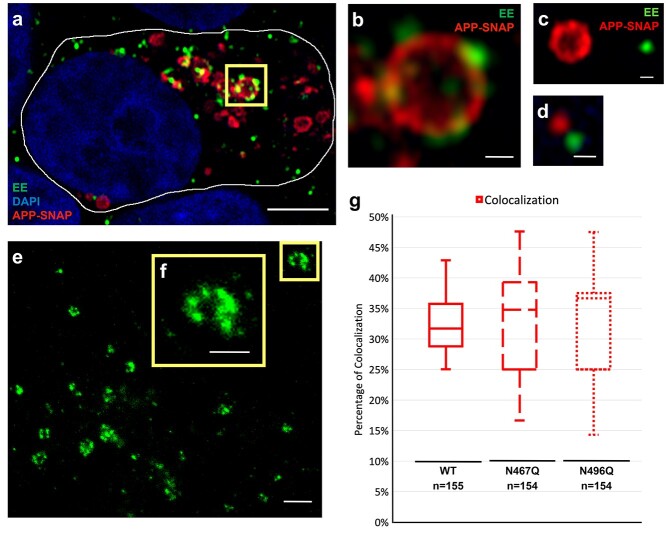
Colocalization of APP-SNAP-positive vesicles with early endosomes in HEK293T cells. To study the presence of the APP variants in early endosomes (EE), HEK293T cells expressing APP-SNAP variants labelled 48 h posttransfection with TMR-star (red) were costained with the early endosomal marker EEA1 (green) and DAPI to visualize nuclei (blue). The samples were analyzed by confocal microscopy with Airyscan detection followed by image analysis. a) Representative image of a cell is shown, with the cell boundary marked by a white line. Scale bar, 5 μm. b) Zoomed-in image of the area inside the yellow rectangle in (a) shows an early endosome that contains APP-SNAP in the endosomal membrane. Scale bar, 500 nm. c and d) Examples of APP containing vesicles that are not labelled with EEA1. Scale bars, 500 nm. e) The morphology of EEA1-positive early endosomes was further characterized by STED microscopy, visualizing patches of EEA1 attached at the membrane of the early endosomes. Scale bar, 1 μm. f) Crop section of the area in the yellow square in (e). Scale bar, 500 nm. g) The proportion of APP-SNAP-positive vesicles that colocalized with the early endosomal marker was analyzed by image analysis of the confocal images. The results are based on at least three independent experiments (each from a different HEK293T cell passage). *N*, number of APP-SNAP-positive vesicles. Statistical analysis was performed using *t*-test with equal variance.

There was an apparent colocalization between APP-SNAP-positive vesicles and early endosomes (Fig. 5a). Within a single HEK293T cell, individual APP-SNAP-positive vesicles of different sizes showed a various degree of colocalization with early endosomes (Fig. 5a–d). To further study the size and morphology of early endosomes in HEK cells, an additional experiment was performed in super-resolution by using stimulated emission depletion microscopy. This experiment showed early endosomes with diameters ranging from 200 to 700 nm containing multiple patches stained by anti-EEA1 antibody (Fig. 5e), which is in agreement with the fact that EEA1 binds to Rab5 on the membrane of early endosomes. Based on data from the airy-scan imaging, a super-resolution detection mode with ~2-fold better resolution than confocal microscopy, ~30% of the APP-SNAP-positive vesicles colocalized with early endosomes. However, there was no significant difference observed between those expressing WT or mutant APP-SNAP (Fig. 5g).

### Lack of glycans leads to reduced levels of lysosomal APP

**Fig. 6:**
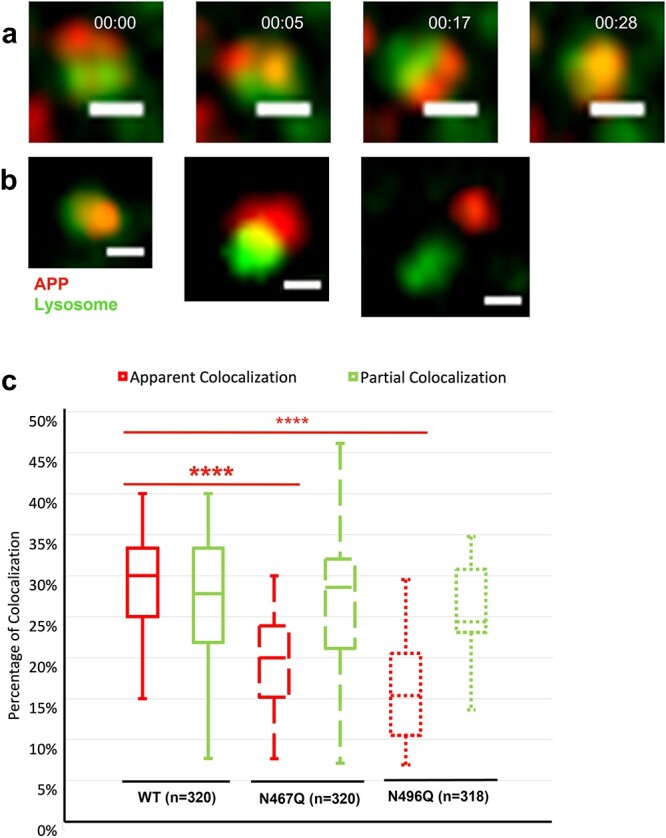
Colocalization of APP-SNAP-positive vesicles with lysosomes in live HEK293T cells. Live cell imaging of APP-SNAP-positive vesicles and lysosomes 48 h posttransfection of HEK293T cells. a) Time lapse of the relative movement between APP-SNAP-positive vesicles and lysosomes. b) Confocal images show the 3 categories of colocalization: apparent colocalization, partial colocalization, and no colocalization. Scale bars: 500 nm. c) Proportion of APP-SNAP-positive vesicles that colocalize with lysosomes. Silicone Rhodamine (SiR)-lysosome kit was used to label live lysosomes and the SNAP-tag of APP was labelled with TMR-star. The number of colocalized APP-SNAP-positive vesicles was measured, calculated in percentage, and plotted. The results were based on at least 3 independent experiments (each from a different HEK293T cell passage) and *N* is the number of APP-SNAP-positive vesicles. Data significance was calculated using *t*-tests with equal variance; ^***^ for 0.0005 < *P* < 0.005; ^****^ for 0.00005 < *P* < 0.0005.

There was also an apparent colocalization between APP-SNAP-positive vesicles and lysosomes. It was shown in live HEK293T cells that APP-SNAP-positive vesicles moved with a fast speed close to and/or within lysosomes over time (Fig. 6a). We then divided these APP-SNAP-positive vesicles into 2 groups: apparent colocalization and partially apparent colocalization (Fig. 6b). In this case, there was a significant decrease in the number of mutant APP-SNAP-positive vesicles that showed an apparent colocalization with lysosomes (Fig. 6c, red).

### Elevated intracellular and secreted Aβ42 levels caused by N-glycosylation mutations on APP-SNAP

**Fig. 7:**
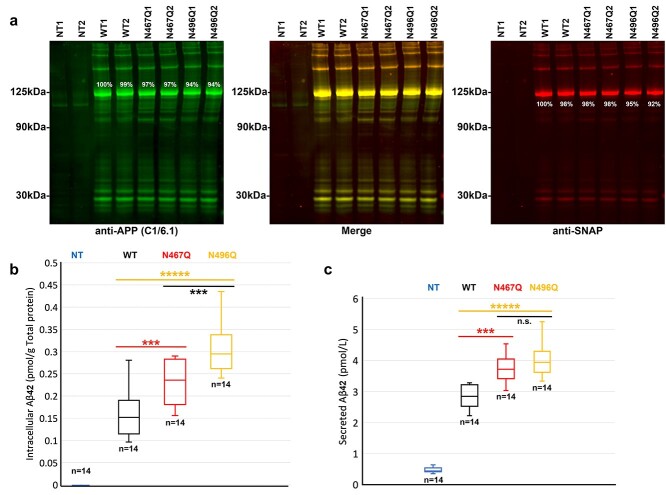
Elevated intracellular and secreted Aβ42 in HEK293T cells expressing APP-SNAP with N-glycosylation mutants. Western blots and Aβ42 ELISA measurements of HEK293T cells 48 h after transfection of APP-SNAP N-glycosylation mutants, WT and NT. a) Gels labeled by anti-APP (C1/6.1) and anti-SNAP-tag antibodies show a relatively equal amount of APP-SNAP expression in all HEK293T cell batches. The intensity of full-length APP band at about 125 kDa was measured and the relative intensity was calculated in percentage against WT1 (white numbers in percentage above or below APP bands). APP-SNAP expression was confirmed with both anti-APP and anti-SNAP-tag antibodies and the endogenous full-length APP expression at about 100 kDa was confirmed by only anti-APP antibody (C1/6.1). b and c) Estimated amount of intracellular (b) and secreted (c) Aβ42 in cell lysate and medium of HEK293T cells, calculated using a standard curve with known levels of Aβ42. The results were based on 4 independent experiments (each from a different HEK293T cell passage) and *N* is the number of independent transfections. Data significance was calculated using *t*-tests with equal variance; ^***^ for 0.0005 < *P* < 0.005; ^*****^ for *P* < 0.00005.

The effects of N-glycosylation mutations on the trafficking and subcellular localization of APP led us to ask the question: will lack of glycosylation affect Aβ production? To answer this question, we measured the levels of intracellular and secreted Aβ42 in HEK293T cells expressing N-glycosylation mutants or WT APP using a human Aβ42-specific ELISA. The amount of Aβ42 produced from endogenous APP in cell lysate was below the limit of quantification, while the level of Aβ42 in the medium was about 0.5 pmol/L. The amount of APP, estimated based on the western blotting, results from 2 samples in each independent experiment (Figs 7a and S3f; between the range from 88% to 116% of WT1), which was similar for the WT and mutant cell lysates. There was a significant increase in the intracellular Aβ42 level in the lysate of cells expressing mutant compared with WT APP-SNAP (Fig. 7b), and the difference between the 2 mutants was also significant. Interestingly, there was also a significant increase in the secreted Aβ42 in the cell culture medium of cells expressing mutant compared with WT APP-SNAP (Fig. 7c).

## Discussion

Previous studies have suggested that the *N*-glycans of APP are implicated in maturation, trafficking, and processing of APP (Tienari et al. 1996; Yazaki et al. 1996; Tsatsanis et al. 2019). Here, we studied the effects of mutations that result in loss of *N*-glycans of APP-SNAP expressed in HEK293T cells, including the effects on Aβ42 generation and secretion.

The expected decrease in molecular weight (~2 kb) of the APP band at ~125 kDa (Fig. 1c and d) in cells expressing N-glycosylation mutants of APP-SNAP is in agreement with removal of 1 *N*-glycan on APP, as previously reported (Påhlsson et al. 1992; Yazaki et al. 1996). The intracellular accumulation of APP species in the soma is in line with previous reports (Tienari et al. 1996; McFarlane et al. 1999) and could be due to a number of reasons. For example, APP trafficking involves the secretory pathway from the Golgi apparatus/*trans*-Golgi network to the plasma membrane, endocytic, and lysosomal pathways between the plasma membrane, endosomes, lysosomes, and the Golgi apparatus/*trans*-Golgi, as well as other less frequent pathways, such as autophagic pathways (Lin et al. 2021). Therefore, the intracellular accumulation of APP in the soma reported here could suggest a delayed sorting, altered, and/or inefficient trafficking of APP. The significant reduction of cell surface APP, as a result of loss of *N*-glycans, could be due to reduced trafficking to the plasma membrane, enhanced processing at the plasma membrane, enhanced endocytosis from the plasma membrane, and/or enhanced trafficking that bypasses the plasma membrane. It has been reported that serum levels of sialyltransferase are significantly decreased in AD patients (Maguire et al. 1994) and cells overexpressing ⍺2,6(N)sialyltransferase showed an increase in the molecular weight of APP species as well as APP secretion, indicating that the presence of terminal sialic acid on complex-type N-linked glycans is required for transport from the Golgi apparatus to the plasma membrane (McFarlane et al. 1999). On the other hand, APP secretion from cells expressing N-glycosylation mutants was not enhanced upon ⍺2,6(N)sialyltransferase overexpression, suggesting that the *N*-glycans on APP are required for the enhanced secretion (Nakagawa et al. 2006).

The wide distribution in the quantification of vesicles could be due to the fact that HEK293T cells multiply rather fast and each HEK293T cell could be at different stage of APP synthesis, processing, and/or trafficking. The decrease in the vesicle size together with the reduction of lysosomal APP in cells expressing mutant APP-SNAP could indicate increased proteolytic processing in other subcellular compartments. Importantly, the altered trafficking we observed in the mutants obviously leads to enhanced amyloidogenic processing, since both intracellular and secreted levels of Aβ42 are significantly increased.

To avoid artefacts associated with too high overexpression, we evaluated the effects of different expression levels on the sizes of endosomal/lysosomal vesicles and confirmed that high expression levels were associated with enlarged sizes of these vesicles, possibly due to the accumulation of APP in lysosomes. Based on these findings, we carefully optimized the amount of APP-SNAP vectors to be introduced into the HEK293T cells and included only cells with moderate expression levels. In previous studies, the amount of APP-coding plasmids was generally over 10 μg. Even if we consider the potentially low transfection efficiency of other transfection methods, they were at least about 20 times over ours. Most likely, high overexpression of APP will alter the cellular processes and thus confound the interpretation of the results.

One way to study effects of glycosylation on cellular processes is to use glycosylation inhibitors. It has been shown that there was a decrease in APP secretion and an increase in intracellular APP when the complex-type N-linked glycosylation was interrupted by mannosidase inhibitor (McFarlane et al. 1999). This accumulation of intracellular APP was present mainly in the perinuclear region, suggesting that APP containing high-mannose core oligosaccharide could be retained in the Golgi apparatus rather than being further trafficked to the plasma membrane (McFarlane et al. 1999). It should be noted, however, that the use of soluble glycosylation inhibitors also influences other cellular components and could thus indicate that defective N-glycosylation of other cellular proteins, and not only APP, could affect the metabolism of APP (Pa 1996; McFarlane et al. 1999). In our study, we introduced the mutation specifically at either of the 2 N-glycosylation sites on hAPP695 and thus studied the role of N-glycosylation on APP in a more specific way. The SNAP-tag enabled us to directly label APP species in live cells with a stable and bright fluorescent signal. To gain further information on the role of N-glycosylation on different APP species, multiple labels could be used in future studies.

To summarize, we have studied the role of N-glycosylation on APP processing and trafficking by generating and expressing 3 APP-SNAP vectors containing WT, N467Q, or N496Q mutant APP-SNAP into HEK293T cells. We have shown that APP-SNAP and its processed CTFs in cells with moderate expression were mainly localized in intracellular vesicles, but also in the Golgi apparatus and the plasma membrane. The replacement of Asn by Gln at either of the 2 N-glycosylation sites of hAPP695 resulted in a reduction in the size of APP-SNAP-positive vesicles, accumulation of APP in the soma, a reduction of APP in the plasma membrane and lysosomes, and elevated levels of intracellular and secreted Aβ42. Altogether, the lack of N-glycosylation of APP leads to altered APP trafficking, resulting in enhanced amyloidogenic APP processing (i.e. Aβ42 formation) and enhanced Aβ42 secretion. These data suggest that N-glycosylation of APP has an implication in AD pathogenesis.

## Materials and methods

### Reagents and materials

Transfection reagent Lipofectamine 3000 (L3000008) was purchased from Thermo Fisher Scientific. Dulbecco’s Modified Eagle’s Medium (41965039, high glucose) was purchased from Thermo Fisher Scientific. Fetal bovine serum (10270-106) was purchased from Thermo Fisher Scientific. Poly-d-lysine precoated 8-well plates (354632) were purchased from Corning. Duolink In Situ Mounting Medium with DAPI (DUO82040) was purchased from Sigma-Aldrich; 10× RIPA buffer (20–188) was purchased from Millipore. γ-Secretase inhibitor L-685,485 (2627) was purchased from Tocris. NuPage 4–12% polyacrylamide Bis-Tris gel (NP0321BOX) and 10× Tris buffered saline (J60764, pH 7.4) were purchased from Thermo Fisher Scientific. Nitrocellulose blotting membranes (10600004) were purchased from GE Healthcare Life Science. Duo prestained protein ladder (928-60000) was purchased from LI-COR Biosciences. Pierce bicinchoninic acid assay (BCA) protein assay kit (23225) was purchased from Thermo Fisher Scientific. Human Amyloid-β 1–42 ELISA kit (27719) was purchased from IBL.

### Construction of APP-SNAP vectors

APP-SNAP plasmids were generated by inserting the hAPP695-SNAP coding sequence [42] into the unique EcoRV site in the pSNAP-tag(m) plasmid (Addgene #101135) containing a CMV promotor. Mutations from original Asparagine (N) to Glutamine (Q) at the 2 known N-glycosylation sites (N467 and N496) of the human WT hAPP695 were inserted by site-directed mutagenesis. All DNA constructs were verified by Sanger sequencing.

### Cell culture

HEK293T cells (Sigma-Aldrich, 12022001-1VL) were seeded in poly-d-lysine precoated 8-well plates 1 day prior to the transfection and cultured in Dulbecco’s Modified Eagle’s Medium with 10% fetal bovine serum. The number of cells seeded was optimized for the length of cell culturing.

### Antibodies and live cell labeling dyes

Primary antibodies: rabbit anti-SNAP-tag antibody (P9310S, 1:250) was purchased from New England BioLabs; C-terminal specific mouse anti-APP (ab32136, clone Y188, 1:5,000) antibody was purchased from Abcam (used for imaging studies and recognizes full-length APP and APP-CTF) C-terminal specific mouse anti-APP (802801, clone C1/6.1, 1:5,000); antibody was purchased from BioLegend.; Alexa Fluor 488 Phalloidin (A12379, 1:200), rabbit anti-EEA1 (F.43.1, 1:200), rabbit anti-Calnexin (PA3-34754, 1:200), and rabbit anti-GRASP65 (PA3-910, 1:200) antibodies were purchased from Thermo Fisher Scientific. Secondary antibodies: IRDye 680RD donkey anti-rabbit (926-68073, 1:15,000) and IRDye 800 CW donkey anti-mouse (92632212, 1:15,000) were purchased from LI-COR Biosciences; goat anti-rabbit Alexa Fluor 647 (A21244, 1:200) was purchased from Thermo Fisher Scientific. Secondary anti-rabbit antibody labelled with Abberior STAR 635P (Sigma-Aldrich) was used for STED microscopy. Live cell labeling dye: SNAP-Cell TMR-Star (S9105S, 1:1,000) was purchased from New England BioLabs; Silicone Rhodamine (SiR)-lysosome kit (SC012, 1:3,000) was purchased from Spirochrome.

### Expression of APP-SNAP plasmid into HEK293T cells

WT and mutant APP-SNAP plasmids were delivered into HEK293T cells with transfection reagent Lipofectamine 3000 according to the manufacturer’s protocol, and the mixtures added directly to the cell culture medium, followed by incubation at 37 °C and 5% CO_2_ for 48 h. The amount of APP-SNAP plasmids introduced into HEK293T cells (0.02–2 μg) was first carefully optimized in order to obtain a sufficient number of HEK293T cells expressing APP-SNAP at a moderate level to avoid artifacts related to overexpression. Thus, DNA concentrations of 0.2–0.5 μg of WT and N467Q/N496Q APP-SNAP vectors were used in the final analysis. The amounts of added plasmids were adjusted to achieve a similar level of expression for APP-SNAP WT and mutants, and with such amount of introduced DNA, there was a low, but quantifiable level of APP-SNAP expression. γ-Secretase inhibitor (L-685,485; 2 μM) was added right after the transfection mixture in some experiments, as indicated. The level of expression was quantified by image analysis and western blot analysis as described below.

### Western blot analysis of APP

HEK293T cells were lysed with 1× RIPA buffer on a 4 °C shaker for 30 min at 48 h posttransfection. The cell lysate was centrifuged at 12,000 × *g* for 20 min at 4 °C to pellet cell debris and the supernatant was collected. The total protein level was measured by BCA. Similar amount of total protein was loaded onto 4–12% polyacrylamide Bis-Tris gels and the individual components of the samples were separated by SDS-PAGE. The separated samples were transferred to nitrocellulose membranes via electroblotting and immunoblotted with anti-APP (Y188) or anti-SNAP-tag antibodies. Relative levels of endogenous APP and APP-SNAP in the cell lysates were then determined by imaging with the OdysseyCLx Imaging System.

Two samples in each ELISA experiment [refer to section Quantification of Aβ levels by enzyme-linked immunosorbent assay (ELISA)] were also analyzed by western blotting, and the intensity of the APP band at about 125 kDa for each sample was measured using Image J/Fiji. The relative amount of APP against WT1 was then estimated in percentage, as shown in Fig. S2i.

### Careful selection of HEK293T cells with a moderate level of expression

The amount of DNA vectors introduced into HEK293T cells was optimized as explained above. About 60–102 HEK293T cells with a moderate level of APP-SNAP expression were carefully selected from at least 3 independent experiments and imaged with confocal microscopes. To be specific, we selected these cells in two steps: (i) first, we selected 6 cells with different level of APP695-SNAP expression and a variety of vesicular sizes. We then measured the total intensity of APP-SNAP, the intensity and number of APP-SNAP-positive vesicles in each of the 4 categories (Fig. S2; 0–0.5, 0.5–1, 1–2, and >2 μm) for each cell. Only those with a moderate level of expression were selected (Fig. S2a–c). (ii) We then randomly selected 10 cells with a moderate level of APP-SNAP expression to confirm the results. In these cells, there was neither saturated level of APP-SNAP nor over-sized APP-SNAP-positive vesicles.

### Cellular labeling of APP-SNAP vectors with SNAP-cell TMR-star

SNAP-Cell TMR-Star stock solution (3 mM) was diluted in cell culture medium to yield a labeling medium containing dye substrate of 3 μM. HEK293T cells were incubated with diluted SNAP-Cell TMR-Star labeling medium at 37 °C and 5% CO_2_ for 30 min, followed by 3 washes with fresh cell culture medium.

### Visualization of APP-SNAP in live and fixed HEK293T cells

Zeiss laser scanning confocal microscopes (LSM980-Airyscan2) or a Nikon A1 laser scanning confocal microscope was used for visualization. Lasers and filters were chosen to illuminate at fluorophore excitation maximum. DAPI channel was used for nucleus, 488-nm channel was used for Alexa Fluor 488, TMR channel (543 nm) was used for SNAP-Cell TMR-Star, and 647-nm channel was used for Alexa Fluor 647. Experimental conditions were kept constant for all experiments.

Leica TCS SP8 STED 3X with Leica microsystems was used for STED imaging, essentially as described previously (Yu et al. 2021). The sample was excited by a 640-nm laser and a 775-nm STED laser was used for emission depletion. Images were taken by a HCPLAPO100x/1.40 oil STED WHITE objective. The region of interest was further zoomed in 4.5 times during acquisition.

### Quantification of Aβ42 levels by ELISA

HEK293T cells from 4 different passages were treated with 14 independent transfection experiments and were lysed with 1× RIPA buffer on a 4 °C shaker for 30 min at 48 h posttransfection. The cell lysate was centrifuged at 12,000 × *g* for 20 min at 4 °C to pellet cell debris and the supernatant was collected. The cell culture medium from each sample was also collected. The amount of total protein in cell lysate and medium was measured by BCA; 100 μl of cell lysate or medium was loaded to ELISA plates precoated with anti-Aβ42 antibody. The amount of human Aβ42 was measured according to the manufacturer’s protocol, calculated from the standard curve for each experiment (Fig. S2c–e), and adjusted to obtain similar level of total protein.

### Image analysis

For size distribution of APP-SNAP-positive vesicles: 151–152 of APP-SNAP-positive vesicles with a moderate level of APP-SNAP expression were selected from at least 3 independent experiments for this analysis. The size of individual APP-SNAP-positive vesicles (diameter; in nm) was measured using Image J/Fiji. These APP-SNAP-positive vesicles were then divided into 4 categories: 0–0.20, 0.21–0.40, 0.41–0.80, and 0.81–1.0 μm, and the size distribution of APP-SNAP-positive vesicles within the selected HEK293T cells was plotted in pie graphs. The differences in the vesicular size were compared between WT and 2 mutants (N467Q and N496Q) and plotted in a box and whisker graph. Data significance was calculated using *t*-test with equal variance.

For determining the extent of APP-SNAP in the plasma membrane: 102 of HEK293T cells with a moderate level of APP-SNAP expression were selected from at least 5 independent experiments. The number of selected cells that contained APP-SNAP in the plasma membrane was firstly counted. Since the fluorescence intensity of APP in the plasma membrane is lower than that in the vesicles, the intensity for visualization of the plasma membrane was increased. Note that the raw data used for calculation are not affected by this procedure; 35.3% (WT), 11.8% (N467Q), and 9.8% (N496Q) of these cells contained APP-SNAP expression in the plasma membrane and this gave us about 10 cells from each variant for further analysis. Ten WT cells were selected in an unbiased manner to match the number of cells in the mutant samples that contained APP-SNAP staining in the plasma membrane. Two parameters in each of the 10 cells were measured separately: (i) total intensity of the whole cell, representing the overall APP-SNAP expression in an individual cell; (ii) intensity of intracellular APP-SNAP, representing the amount of intracellular APP-SNAP expression of each cell. The amount of APP-SNAP in the plasma membrane was calculated by subtracting (ii) from (i), and the results were then plotted in a box and whisker graph. Data significance was calculated using *t*-tests with equal variance.

For colocalization of APP-SNAP-positive vesicles with early endosomes: 154–155 of APP-SNAP-positive vesicles with a moderate level of APP-SNAP expression were selected from at least 3 independent experiments. The number of APP-SNAP-positive vesicles with or without colocalization with early endosomes was measured and calculated in percentage. The percentage of colocalization was then plotted in a box and whisker graph. Data significance was calculated using *t*-tests with equal variance.

For colocalization of APP-SNAP-positive vesicles with lysosomes: 318–320 of APP-SNAP-positive vesicles with a moderate level of APP-SNAP expression were selected from at least 4 independent experiments. APP-SNAP-positive vesicles were firstly divided into 2 categories: apparent colocalization and partial colocalization. The number of APP-SNAP-positive vesicles with or without colocalization with lysosomes was measured and calculated in percentage. The percentage of colocalization was then plotted in a box and whisker graph. Data significance was calculated using *t*-tests with equal variance.

For measurement of Aβ42: The amount of intracellular and secreted Aβ42 was measured separately in cell lysate and medium, calculated using the standard curve from each experiment, and plotted in a box and whisker graph. The result was based on 4 independent experiments from 4 different HEK293T passages, and each sample was transfected individually. Data significance was calculated using *t*-tests with equal variance.

## Supplementary Material

Supplementary_Information_220216_final_revised_cwac009

Video_S1_WT_cwac009

Video_S2_N467Q_cwac009

Video_S3__496Q_cwac009

## Abbreviations

Aβ, amyloid-β peptide; AD, Alzheimer disease; AICD, APP intracellular domain; APP, amyloid precursor protein; BCA, bicinchronic acid assay; CTF, C-terminal fragment; EEA1, early endosome antigen 1; ELISA, enzyme-linked lectin assay;, hAPP, human APP;HEK, human embryonic kidney; N, asparagine; NT, non-transfected; Q, glutamine; sAPP, soluble APP;SiR, silicone rhodamine; STED, stimulated emission depletion; WT, wildtype
