# Supplementary material for: Lack of N-glycosylation increases amyloidogenic processing of the amyloid precursor protein
Source: Glycobiology. 2022 Mar 10;32(6):506–17. doi: 10.1093/glycob/cwac009 (PMC9132248; doi:10.1093/glycob/cwac009)
Supplement: Supplementary_Information_220216_final_revised_cwac009 [file supplementary_information_220216_final_revised_cwac009.pdf]

Supplementary Information

Supplementary Figures

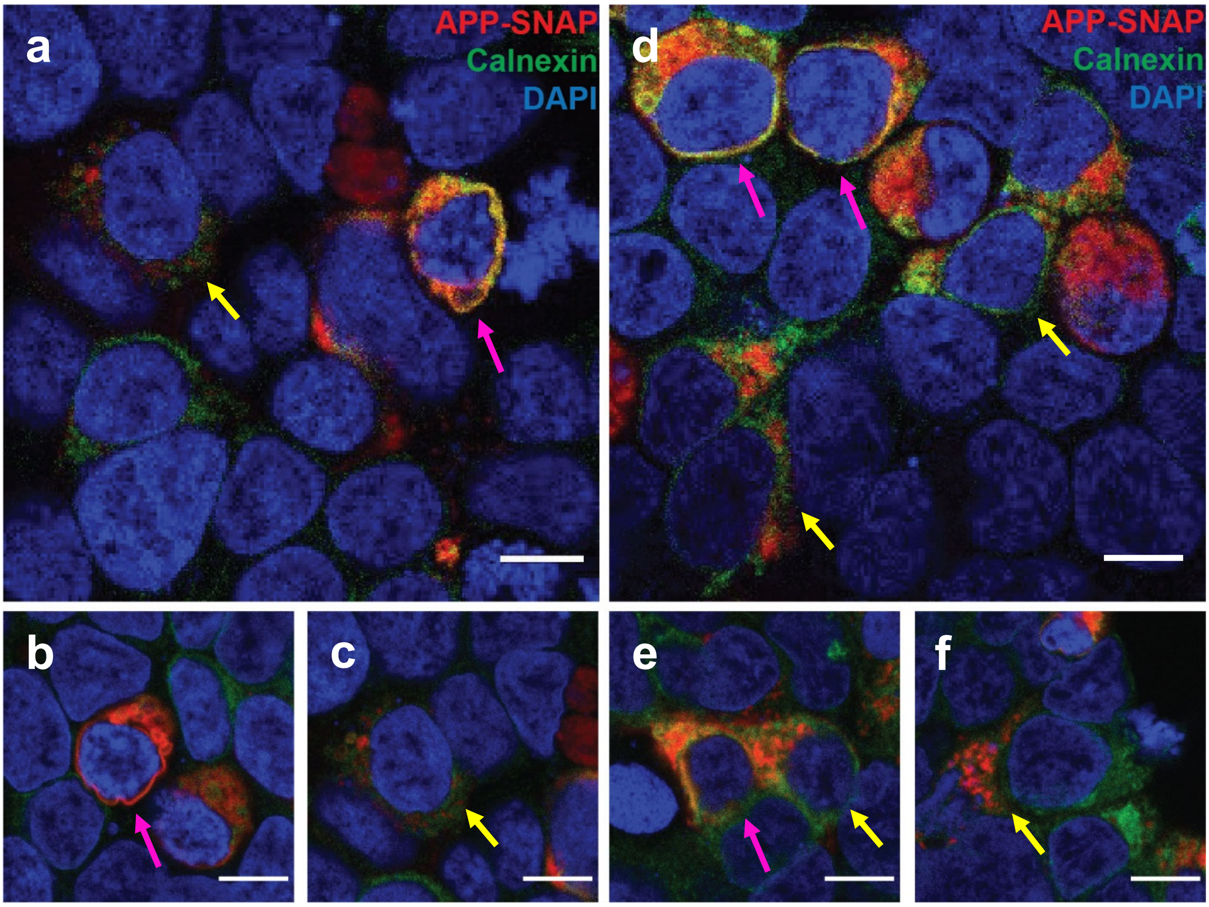

Figure S1. Retention of hAPP695-SNAP variants caused by overexpression.

Confocal images of HEK2293T cells expressing hAPP695-SNAP variants with variable degree of expression levels. Representative images are shown for WT (a-c) and the N-glycosylation mutant 467Q (d-f). ER was stained by an anti-Calnexin antibody (green), APP-SNAP was stained by TMR-star (red) and nuclei were stained by DAPI (blue). Images were taken by 60X oil immersion objective without cropping (a, d) or with 5-fold cropping (b, c, e, f). Yellow arrows point at cells with low to moderate expression, Magenta arrows point at cells with high moderate expression. Scale bars, 10 µm.

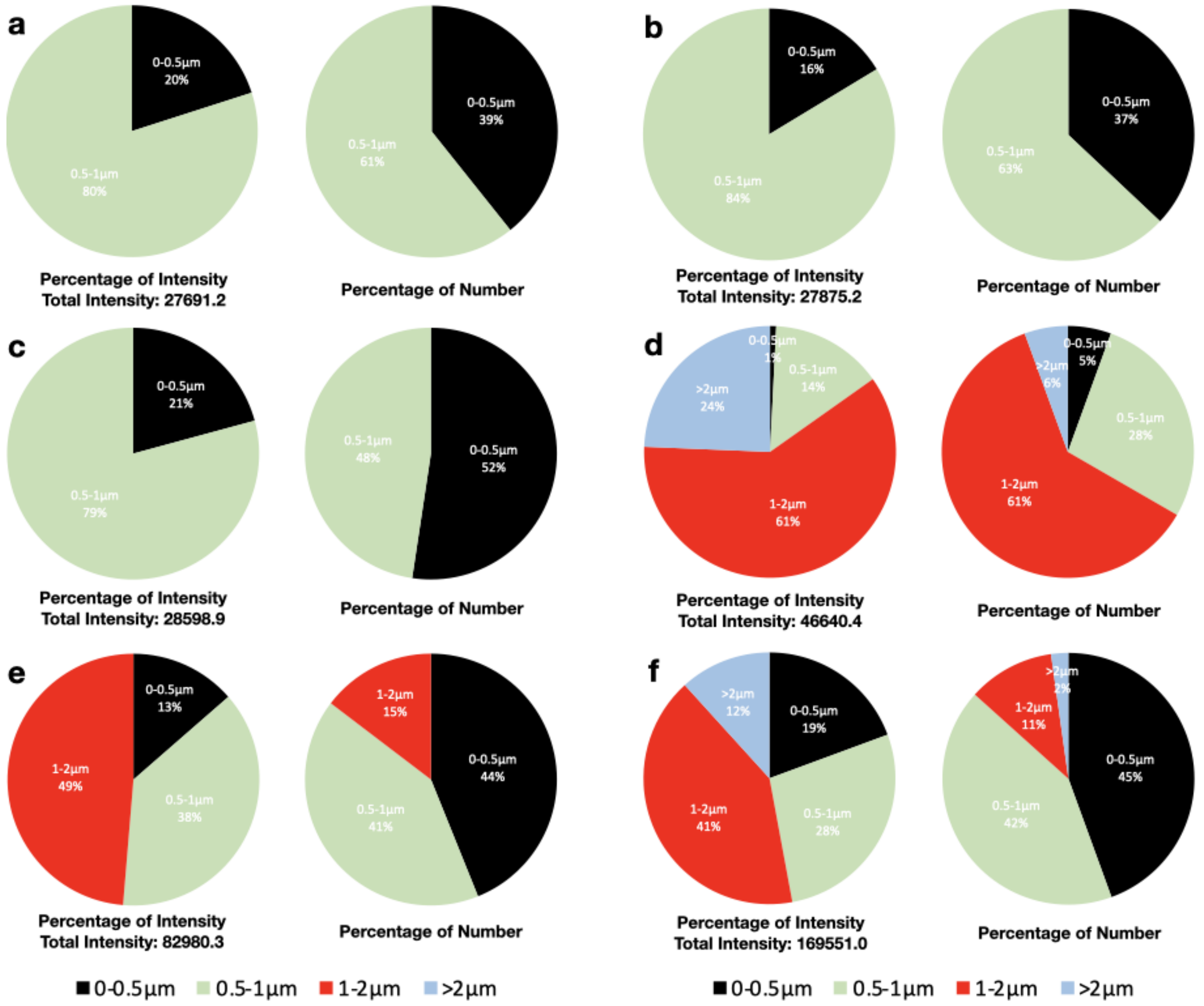

Figure S2. Analysis of the effect of the expression level on the size of APP-containing vesicles. In the first step of the selection, six HEK293T cells with different level of hAPP695-SNAP expression and a variety of vesicular size were selected. hAPP695-SNAP-positive vesicles were divided into four groups based on their diameters: 0-0.5 μm, 0.5-1 μm, 1-2 μm and >2 μm. The total intensity of hAPP695-SNAP expression within the whole cell, the total intensity of hAPP695-SNAP-positive vesicles (left) as well as the number of hAPP695-SNAP-positive vesicles (right) in each of the four categories were measured, calculated and plotted in percentage. (a-f) Six representative HEK293T cells. Cells containing saturated level of hAPP695-SNAP expression and/or over-sized hAPP695-SNAP-positive vesicles (d-f) were excluded from further analysis.

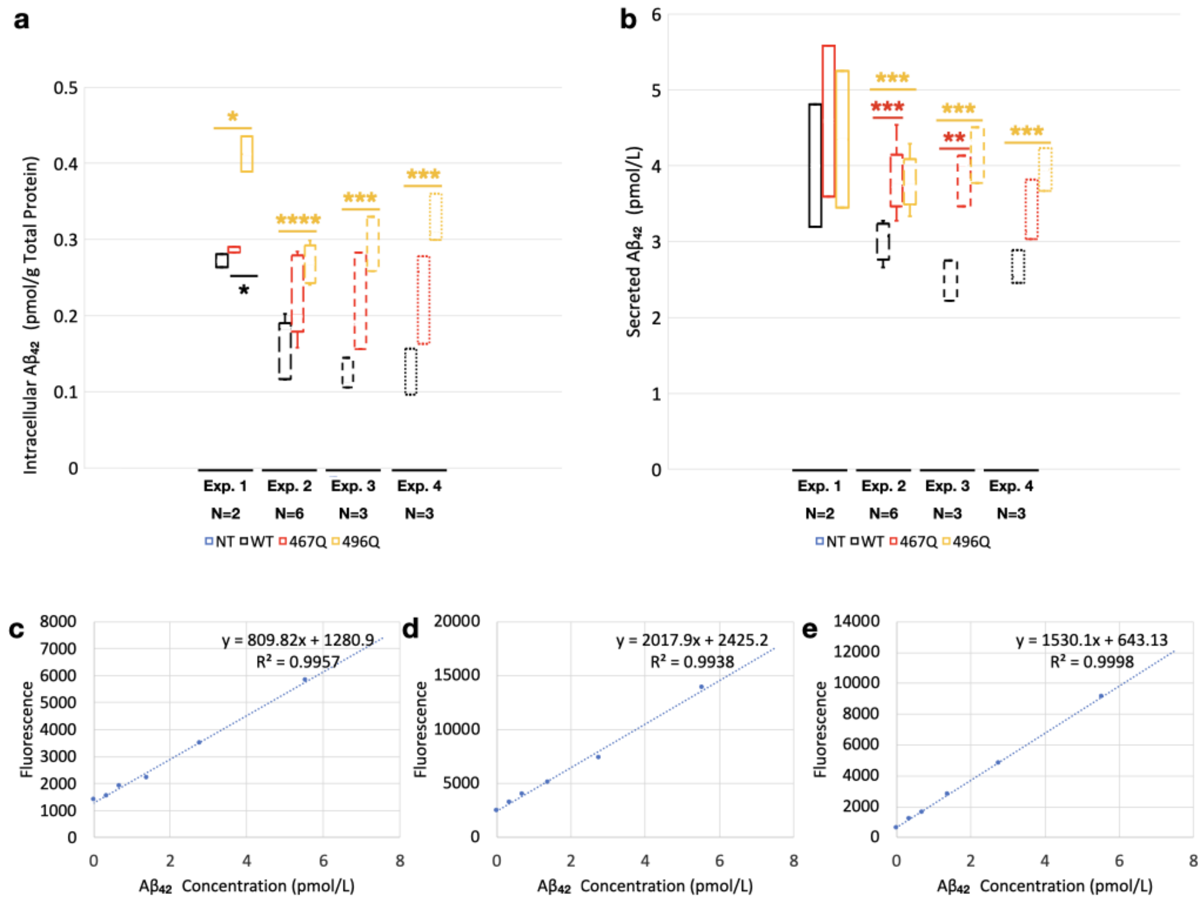

f	Relative Intensity of AβPP (%) by Anti-AβPP Antibody			Relative Intensity of AβPP (%) by Anti-SNAP-tag Antibody		
	Experiment 1	Experiment 2	Experiment 3-4	Experiment 1	Experiment 2	Experiment 3-4
WT1	100	100	100	100	100	100
WT2	112	99	95	102	98	92
467Q1	107	97	116	96	98	114
467Q2	97	97	112	92	98	109
496Q1	101	94	99	94	95	97
496Q2	96	94	92	88	92	88

Figure S3. Data for ELISA performed in HEK293T cells.

(a-b) Box and whisker graphs show the estimated amount of intracellular (a) and secreted (b) Aβ42 in cell lysate and medium of HEK293T cells. The amount of intracellular and secreted Aβ42 was measured separately in cell lysate and medium, calculated using each standard curve and plotted in a box and whisker graph. The results were based on four independent experiments (each from a different HEK293T cell passage) and N is the number of independent transfections. Data significance was calculated using t-tests with equal variance; *** for $0.0005<p<0.005$; ***** for $p<0.00005$. (c-d) Standard curves for experiments 1-4. Experiment 1 was calculated using standard curve in (c) and experiment 2 was calculated using standard curve in (d). Experiments 3 and 4 were measured in the same plate and were calculated using the same standard curve in (e). (f) Table shows the relative intensity of AβPP labeled by anti-AβPP or anti-SNAP-tag antibodies. Two samples of WT, N467Q and N496Q from each

experiment was quantified with SDS-PAGE. The intensity of AβPP bands at about 125kDa was measured and the relative amount of AβPP in each sample calculated in percentage against WT1 (ranged from 88% to 116% of WT1).

Supplementary Videos

Video S1. Live cell imaging of WT APP-SNAP-positive vesicles and lysosomes in HEK293T cells. Live cell imaging of WT hAPP695-SNAP-positive vesicles and lysosomes 48h post transfection of HEK293T cells. Time lapse showing the movement between hAPP695-SNAP-positive vesicles and lysosomes. Silicone Rhodamine (SiR)-lysosome kit was used to label live lysosomes and the SNAP-tag of APP was labelled with TMR-star. Imaging was performed by a confocal microscope equipped with Airyscan detector.

Video S2. Live cell imaging of N467Q APP-SNAP-positive vesicles and lysosomes in HEK293T cells. Live cell imaging of N467Q hAPP695-SNAP-positive vesicles and lysosomes 48h post transfection of HEK293T cells. Silicone Rhodamine (SiR)-lysosome kit was used to label live lysosomes and the SNAP-tag of APP was labelled with TMR-star. Imaging was performed by a confocal microscope equipped with Airyscan detector.

Video S3. Live cell imaging of N496Q APP-SNAP-positive vesicles and lysosomes in HEK293T cells. Live cell imaging of N496Q hAPP695-SNAP-positive vesicles and lysosomes 48h post transfection of HEK293T cells. Time lapse showing the movement between hAPP695-SNAP-positive vesicles and lysosomes. Silicone Rhodamine (SiR)-lysosome kit was used to label live lysosomes and the SNAP-tag of APP was labelled with TMR-star. Imaging was performed by a confocal microscope equipped with Airyscan detector.
